# Tropism and Retinal Transduction Efficiency of Adeno-Associated Virus Serotypes in Mice

**DOI:** 10.1167/iovs.66.12.18

**Published:** 2025-09-08

**Authors:** Wenjing Wu, Joel McRae, Asa Brown, Martin-Paul Agbaga

**Affiliations:** 1 Department of Ophthalmology, Dean McGee Eye Institute, University of Oklahoma Health Sciences Center, Oklahoma City, Oklahoma, United States; 2 Department of Cell Biology, University of Oklahoma Health Sciences Center, Oklahoma City, Oklahoma, United States

**Keywords:** photoreceptor cells (PRs), retinal pigment epithelial cells (RPEs), subretinal injections, intravitreal injections, adeno-associated virus (AAV)

## Abstract

**Purpose:**

Adeno-associated viruses (AAVs) have become the preferred vector for gene therapy in ophthalmology. However, requirements for specific cell surface receptors limit AAV-mediated retinal cell transduction efficiency. This led to the need to engineer novel AAV vectors for widespread retinal transduction and transgene expression. However, no comparative analyses of these novel AAV serotypes have been reported. Here, we compare the retinal transduction efficiency of four novel AAV serotypes in wild-type mice retina after intravitreal and subretinal injections.

**Methods:**

In total, 1.0 µL each of the four different AAV/cytomegalovirus/enhanced green fluorescent protein (EGFP) synthetic serotypes (1 × 10^9^ genome copies [GC]/eye) was delivered by intravitreal or subretinal injection into mouse eyes. Tropism of each serotype to efficiently transduce photoreceptor (PR) and retinal pigment epithelial (RPE) cells was examined by EGFP expression using fundoscopy and immunolabeling 1 and 2 months after administration. Retinal function was evaluated using electroretinography and optomotor kinetics.

**Results:**

Fundoscopy and immunolabeling of EGFP in both subretinally and intravitreally injected AAV/DJ and AAV/DJ8 retinas showed the highest transduction efficiency. Compared to intravitreal delivery, all serotypes successfully transduced PR and RPE cells after subretinal injections. However, only intravitreally delivered AAV27m8, AAV/DJ, and AAV/DJ8 efficiently transduce PRs. AAV/DJ8 exhibited the highest PR and RPE transduction of the four serotypes. Visual function was unaffected, and adverse immunologic responses were not observed between the serotype and the PBS-injected eye.

**Conclusions:**

Synthetic AAV serotypes differentially transduced PR and RPE cells depending on the delivery route. AAV/DJ8 exhibited the most efficient transduction of PR and RPE cells when injected intravitreally.

Mutations in over 270 genes have been identified as causes of retinal degeneration (RD) in humans.^1^ These mutations typically lead to the progressive death of rod and/or cone photoreceptors (PRs) and retinal pigment epithelium (RPE) cells (https://sph.uth.edu/retnet/).^2^ Currently, there is only one therapy approved by the US Food and Drug Administration (FDA) for a small subset of patients with RD with limited efficacy, highlighting a critical need for innovative treatment strategies to preserve vision loss associated with these genetic mutations. Gene therapy has emerged as a promising therapeutic approach to address the genetic basis of RD.^3^ The strategy involves introducing the functional copy of the mutated gene or a gene-corrective mechanism into the host's target cells to modify the defective DNA and alleviate its pathological consequences, ultimately achieving a therapeutic outcome.^4^

Adeno-associated viral (AAV) vectors have become a powerful tool for gene delivery due to their broad host tropism, low immunogenicity, and ability to provide long-term gene expression in the nondividing retinal cells. Recombinant AAVs (rAAV) are engineered by replacing the viral genome with the desired therapeutic gene while preserving the viral inverted terminal repeats necessary for packaging the transgene into functional viral particles.^5^ In 2017, the FDA approved LUXTURNA, the first gene therapy derived from naturally occurring AAV2, for treating RPE65-associated Leber congenital amaurosis. This breakthrough sparked excitement about AAV-based therapies as a potential cure for RD.^6^ However, the use of these first-generation AAV vectors has revealed significant challenges, including the presence of host-neutralizing antibodies, poor transduction efficiency across the entire retina, and suboptimal cell-specific transgene expression, which continue to limit treatment efficacy.^7^^,^^8^ It has been known since the 1970s that neutralizing antibodies against AAV2 are prevalent in the human population, with a seroprevalence of approximately 80%.^9^ Thus, engineered novel AAVs with limited human exposure and fewer neutralizing antibodies could enhance the long-term success of gene therapy strategies.

Another challenge of AAV-mediated gene therapy is the complexity of delivering treatment to the PR and RPE cells at the outer retina. Therapeutic agents are typically administered through subretinal or intravitreal injections to target these retinal cells.^10^ Subretinal injection involves delivering the therapeutic agents into the subretinal space between the RPE and the PR. Intravitreal injections involve delivery into the vitreous humor near the inner retina and ganglion cells. While subretinal delivery is effective, it requires a more invasive surgical procedure and carries risks such as secondary retinal atrophy, retinal detachment, and retinal hemorrhages.^11^ Likewise, in research animal models, subretinal injections often result in retinal detachment and bleeding.

In contrast, intravitreal injections are less invasive and can be performed in a clinical setting.^12^ Ideally, this method could target the outer retina. However, in practice, it faces challenges in reaching the outer retina due to the inner limiting membrane (ILM), an extracellular matrix layer that separates the retina from the vitreous and acts as a barrier. Composed of tightly packed collagen, laminin, and negatively charged glycoproteins, the ILM restricts the passage of negatively charged viral vectors, limiting their effectiveness in reaching the outer retina.^13^

Recent advancements in vector engineering, through rational design and in vivo–directed evolution, have led to the development of a wide array of novel synthetic AAV vectors.^14^ These new vectors address the challenges of delivering treatments to the outer retina via intravitreal injection, overcoming the limitations of first-generation AAV vectors.^14^^,^^15^ Innovations in capsid and transgene modifications have significantly improved AAV transduction efficiency and cell-specific expression in retinal tissues. Notably, several newly developed third-generation AAV vectors, including AAV2^QYF^, AAV2^7m8^, AAV/DJ, and AAV/DJ8, have demonstrated pan-retinal transduction and effective infection of the outer retina in various preclinical models when delivered intravitreally.^14^^,^^16^^–^^21^ Boye et al.^22^ reported that AAV2^QYF^, which was engineered to contain four tyrosine-to-phenylalanine (Y-F) mutations in the AAV2 capsid to enhance the vector's resistance to intracellular proteasomal degradation, improved retinal transduction and transgene expression efficiency when injected intravitreally. AAV2QYF has been used in clinical trials for achromatopsia (NCT02935517 and NCT02599922). AAV2^7m8^ has a 10-amino-acid insertion in the heparan-binding region of the AAV2 capsid and was selected for its reported superior performance compared to AAV2.^16^ The engineered AAV capsid, AAV2^7m8^, is considered among the best capsids for transducing the retina following intravitreal injection.^23^ Thus, it has been used in several clinical studies in the eye, specifically for conditions like AMD (NCT03748784) and RP (NCT03326336).^24^ The AAV/DJ and its advanced version, AAV/DJ8, optimized using properties of AAV8 serotypes, have shown promising results and strong preclinical efficacy, particularly in transducing various retinal cell types with both intravitreal and subretinal injections, outperforming AAV2.^7^^,^^18^ However, there are no published reports on AAV/DJ8 efficiency in transducing retinal cells in vivo, except it has been predicted to target several organs by commercial entities like Addgene and Vector Builder. However, no prior studies have compared the retinal transduction efficiency of these serotypes side by side when delivered by intravitreal and subretinal injections.

Here, we determined the retinal transduction efficiency and transgene expression characteristics of four different AAV serotypes following subretinal or intravitreal injections in adult mouse eyes using a noninflammatory viral titer concentration of 1 × 10^9^ GC/eye.^25^ We demonstrated how the AAV serotypes differentially transduce PR and RPE cells based on the delivery route. Our results identified AAV/DJ8 as the most efficient serotype for transduction of PR and RPE cells when delivered intravitreally. These findings will guide researchers in retinal gene therapy studies to achieve high retinal transduction efficiency through intravitreal injections.

## Methods

### AAV Serotypes and Vector Construct

The four AAV serotypes (AAV2^QYF^, AAV2^7m8^, AAV/DJ, and AAV/DJ8) were selected based on previous reports of their excellent transduction of the retinal cells at high titers.^14^^,^^16^^–^^19^^,^^26^ These vectors were designed and obtained from VectorBuilder (Chicago, IL, USA). The recombinant AAV constructs included a cytomegalovirus-driven enhanced green fluorescent protein (EGFP) transgene cassette with the Woodchuck Hepatitis Virus Posttranscriptional Regulatory Element to enhance transgene expression. Each serotype was used at a working viral titer of 1 × 10^9^ GC/eye.

### Experimental Animals

All animal experiments adhered to the ARVO statement for using animals in ophthalmic and vision research and were approved by the Institutional Animal Care and Use Committee of the University of Oklahoma Health Sciences Center (Oklahoma City, OK, USA). C57BL/6J (both sexes, postnatal day 60) from Jackson Laboratory (Bar Harbor, ME, USA) were used. The animals were genotyped for known RD-causing variants. Animals were maintained on a 12-hour light/dark cycle, with food and water ad libitum.

### Intraocular Injections of AAV

Postnatal day 60 (P60) C57BL6J mice, both males and females, were anesthetized with an intraperitoneal injection (IP) of ketamine (100 mg/kg)/xylazine (10 mg/kg), followed by corneal application of 0.5% proparacaine. Pupils were dilated with 1% tropicamide and 2.5% phenylephrine HCL (Akorn Pharmaceuticals, Lake Forest, IL, USA). Injections were performed using a 10-µL nanofil injector system with a 33-gauge blunt needle (World Precision Instruments, Sarasota, FL, USA). A trans-scleral puncture was made with a 25-gauge needle behind the limbus under an ophthalmic surgical microscope (Carl Zeiss Surgical, Thornwood, NY, USA). For intravitreal injections, a 33-gauge blunt needle was inserted through the puncture and advanced to the central retina to deliver 1 µL of viral particle (1 × 10^9^ GC/eye). For subretinal injections, the needle was further extended through the retina to inject into the subretinal space. The needle was held in place for 10 to 15 seconds before it was withdrawn to avoid flushing back. Erythromycin ophthalmic ointment (Perrigo, Minneapolis, MN, USA) was applied to prevent infection.

### Fundoscopic Analysis

A Phoenix Micron IV Retinal Imaging system (Phoenix Research Labs, Pleasanton, CA, USA) was used to visualize retinal EGFP transgene expression in AAV-injected mice. Mice were anesthetized with 4% isoflurane and maintained with 3% isoflurane via a nosecone, and pupils were dilated using 1% tropicamide and 2.5% phenylephrine HCL (Akorn Pharmaceuticals). After dilation, artificial tears (Systane Ultra; Alcon, Fort Worth, Texas, USA) were applied for lubrication. Fluorescent imaging was conducted using an EGFP excitation light source and detected with a green light filter, maintaining consistent gain settings. Fluorescence intensity was analyzed using ImageJ Fiji software (National Institutes of Health, Bethesda, MD, USA).

### EGFP Immunofluorescence Analysis

Mice were euthanized with CO_2_, their eyes were enucleated, and the anterior chamber was removed to obtain the posterior eye tissue. For whole mounts, the retina and eyecup were fixed in 4% paraformaldehyde overnight at 4°C, rinsed in PBS three times, and blocked in blocking buffer (0.1% Triton X-100 and 5% bovine serum albumin in PBS), followed by incubation in primary antibodies overnight at 4°C and secondary antibodies for 2 hours at room temperature. The eyecup and retina were flattened on a slide to form a flower-like structure before mounting.

For cryosectioning, eye cups (retina, RPE, and sclera layers) were infiltrated with 30% sucrose and embedded in a tissue-freezing medium (O.C.T.-TEK; Sakura, Torrance, CA, USA). Approximately 100 sections, each 10 µm thick, were cut along the horizontal plane. Every third section was taken and placed onto microscope slides, with sections representing the whole eye at different levels. EGFP expression was detected using anti-EGFP antibody, followed by a counterstain with 4′, 6-diamidino-2-phenylindole (DAPI; Life Technologies, Waltham, Massachusetts, USA), and then mounted with Vectashield antifade media (Vector Laboratories, Burlingame, CA, USA). Fluorescence images were captured using a laser-scanning confocal microscope (FV 1200; Olympus, Center Valley, PA, USA).

### Western Blot Analysis

Western blot analysis was performed as described previously.^27^ Briefly, the retinas or eyecups were homogenized in RIPA buffer containing protease inhibitors. Homogenates were centrifuged at 10,000 × *g* for 10 minutes, and the supernatant was collected. Protein concentrations were determined with the BCA assay kit (Pierce BCA Thermo Fisher, Waltham, Massachusetts, USA). A total of 20 µg of protein was resolved on 10% SDS-PAGE, transferred to a nitrocellulose membrane, and blotted with the indicated antibodies. Band densitometry analysis was performed using ImageJ (National Institutes of Health). A total of six retinas or eyecups from each serotype were pooled. Statistical analysis was performed over technical repeats.

### Histologic Analysis

For paraffin sections, enucleated eyes were fixed in Davidson's fixative (20 mL of 10% neutral buffered formalin, 10 mL of glacial acetic acid, 30 mL of 95% (v/v) ethanol, and 30 mL of distilled water) for 36 hours and transferred to 70% EtOH. The eyes were dehydrated in graded ethanol, permeabilized with xylene, and embedded in paraffin, as previously described.^27^ Sections were cut at a 5-µm thickness using a microtome (Leica RM2235; Leica Microsystems, Bensheim, Germany) and annealed onto glass slides at 60°C. Hematoxylin and eosin staining was performed to assess retinal morphology, and images were captured using a Motic slide scanner (EasyScan One; Motic, Kowloon, Hong Kong).

### Electrophysiological Recordings

Mice were dark-adapted overnight (∼16 hours) and anesthetized under dim red light by an IP injection of ketamine (100 mg/kg)/xylazine (10 mg/kg). Pupils were dilated with topical 1% tropicamide and 2.5% phenylephrine HCL (Akorn Pharmaceuticals), and gel corneal lubricant (Systane Ultra; Alcon) was applied to keep the corneas hydrated. The body temperature was maintained at 37°C with a heating pad. Electroretinogram (ERG) responses were recorded using the Espion E3 machine with a Ganzfeld Color Dome system (Diagnosys LLC, Lowell, MA, USA). Retina voltage changes to light were recorded from both eyes using gold loop electrodes (Diagnosys LLC). The reference electrode was placed subcutaneously into the forehead, and the ground electrode was inserted subcutaneously into the tail. Full-field ERGs were performed using flashes of increasing light intensities. Mice were allowed to re-dark adapt (2–5 minutes) between flashes to recover from photobleaching.

### Optomotor Response Assessment

Vision in AAV-injected mice was assessed using the OptoMotry-AT system (Cerebral Mechanics, White Plains, NY, USA), as previously described.^28^ Briefly, mice were placed on a platform in a virtual reality chamber with four monitors displaying sine wave gratings rotating at 12 deg/s. A video camera tracked the mice, and the optomotor reflex was assessed automatically. To evaluate visual acuity, the grating started at a spatial frequency of 0.042 cyc/deg and gradually increased until the head-turning response was no longer observed. The spatial frequency threshold was recorded at 100% contrast.

Antibodies included MS anti-GFAP CHEMICON MAB360 (Southfield, Michigan); Rb anti–Glutamine Synthetase Proteintech 11037-2-AP (Rosemont, Illinois); MS anti-EGFP Proteintech 66002-1-Ig; MS anti-Rhodopsin 1D4 Invitrogen MA1-722 (Carlsbad, California); MS anti-GAPDH Proteintech 60004-1-Ig; Rb anti-RPE65 Proteintech 17939-1-AP; Donkey anti-Rabbit Alexa Fluor-594 Invitrogen A32766; Donkey anti-Mouse Alexa Fluor-488 Invitrogen A32790; Donkey anti-Mouse Alexa Fluor-647 Invitrogen A32787TR647; WGA-Fluorescein Vector Laboratorios FL-1021 (Neward, California); HRP-conjugated Goat Anti-Mouse Proteintech SA00001-1; and HRP-conjugated Goat Anti-Rabbit Proteintech SA00001-2.

### Statistical Analysis

Statistical analyses were performed using Prism software (GraphPad, La Jolla, CA, USA). A *P* value of less than 0.05 was considered statistically significant. An unpaired *t*-test was used for comparisons between the two groups. One-way ANOVA, followed by Tukey's post hoc test, was used for multiple variance analyses.

## Results

### Retina Structure and Transgene Expression in the AAV-Injected Wild-Type Mice

**Figure 1.**
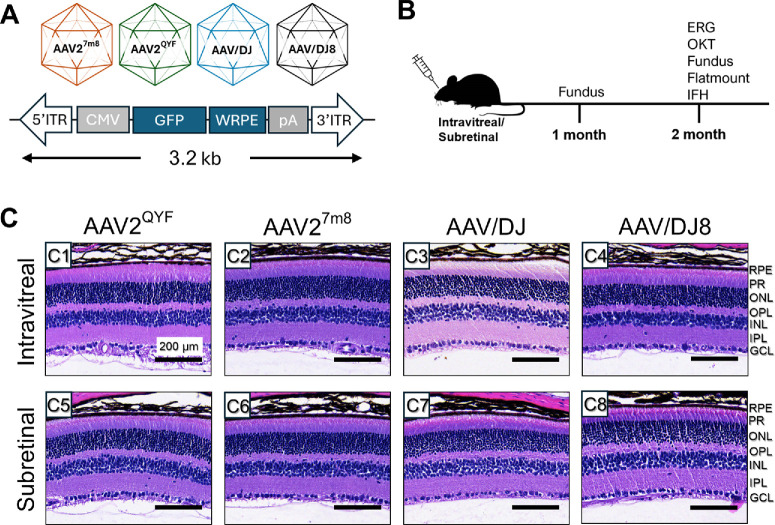
Preserved retina morphology was observed in both intravitreally and subretinally injected mouse eyes. (**A**) Schematics of the rAAV construct and the capsid of various serotypes used in this study. All serotypes are based on an AAV/cytomegalovirus (CMV)/EGFP construct with a Woodchuck Hepatitis Virus Posttranscriptional Regulatory Element cassette, which drives ubiquitous expression of EGFP under the control of a CMV promoter. (**B**) Schematic of the study timeline with assays performed at each time point. Histologic evaluation of AAV-injected retinas used in the current study at 8 weeks postinjection. (**C**) Representative images of mid-peripheral retinal sections from AAV-injected retinas via (C1–C4) intravitreal and (C5–C8) subretinal methods stained with hematoxylin and eosin. GCL, ganglion cell layer; INL, inner nuclear layer; IPL, inner plexiform layer; IS, photoreceptor inner segments; ONL, outer nuclear layer; OPL, outer plexiform layer; OS, photoreceptor outer segments. *n* = 4 eyes/group.

**Figure 2.**
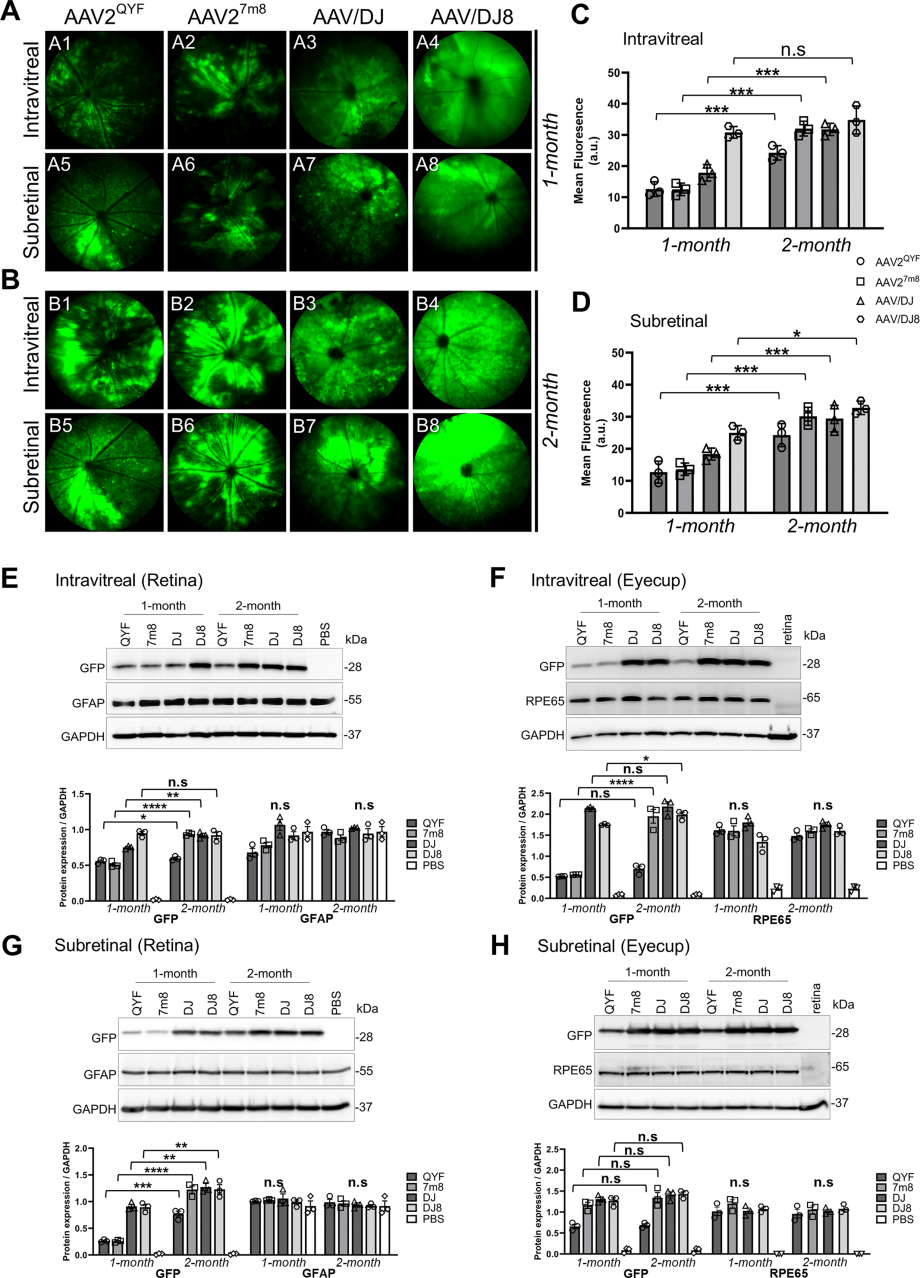
Intravitreal and subretinal delivery of AAV serotypes exhibited a progressive increase in EGFP expression in the retina. Representative fundus photographs show EGFP expression in live mouse eyes at 1-month (**A**) and 2-month (**B**) postinjection time points. Quantification of EGFP fluorescence intensity in the 1-month postinjected (**C**) and 2-month postinjected (**D**) mice eyes, *n* = 3 mice/group. Western blot analysis of EGFP protein expression in the 1-month and 2-month intravitreal (**E**, **F**) and subretinal (**G**, **H**) injected mouse retina and eyecups. Results presented as the mean ± SD (one-way ANOVA with Tukey's correction, *P < 0.5, **P < 0.01, ***P < 0.001, ****P < 0.0001). Statistical analysis was averaged from three technical repeats of six pooled retinas or eye cups from each serotype.

Four AAV serotypes, AAV2^QYF^, AAV2^7m8^, AAV/DJ, and AAV/DJ8, were compared for their retinal transduction efficiency following intravitreal or subretinal delivery. Retinal structural integrity and in vivo transgene expression were evaluated in C57BL6J mice (WT) 2 months postinjection (Figs. 1A, 1B). Subretinal injections can often lead to vasculature damage, bleeding into the eye, and sometimes cataracts. Therefore, 1 month after injection, fundus imaging was done, and animals with healthy retinal layers and no cataracts were selected for further analyses. Hematoxylin and eosin staining was performed at the 2-month mark to assess retinal structural integrity (Fig. 1C). Concurrently, in vivo EGFP expression was imaged using a Micron IV funduscope with a UV filter. Among the four rAAV vectors, AAV/DJ8, regardless of the delivery method, showed the strongest in vivo EGFP fluorescence intensity at 1 and 2 months postinjection (Figs. 2A4, 2B4). For each serotype, EGFP fluorescence increased over time in both intravitreal and subretinal injected eyes. The one exception was AAV/DJ8, which had already reached its highest fluorescence at 1 month postinjection when delivered intravitreally, demonstrating the quickest and most efficient retinal transduction and transgene expression (Fig. 2C).

Western blot analysis of the AAV-injected retinas supported our in vivo fundus findings. AAV/DJ8 reached the highest expression of EGFP at 1 month in retinas injected intravitreally (Fig. 2E), with levels remaining stable thereafter, while the other serotypes showed a gradual increase over the same period. In intravitreally injected eyecups (Fig. 2F), expression of EGFP in both AAV/DJ and AAV/DJ8 peaked at 1 month, while AAV2^7m8^ increased in EGFP expression. This indicates that AAV/DJ and AAV/DJ8 penetrated the outer retina more effectively than AAV2^7m8^ and AAV2^QYF^.

In subretinally injected retinas, AAV/DJ and AAV/DJ8 showed robust EGFP expression at 1 month, with gradual increases observed by 2 months (Fig. 2G), suggesting a slow spread of AAV particles from the subretinal space into the retina. All subretinally injected AAV particles mediated EGFP expression in the RPE in the eyecups (Fig. 2H), although AAV2^QYF^ had the lowest expression. Interestingly, there was no increase in EGFP expression in all four serotypes, suggesting no additional viral movement occurred after the 1-month injection point.

### Outer Retina Transgene Coverage in the AAV-Injected WT Mice Eye

**Figure 3.**
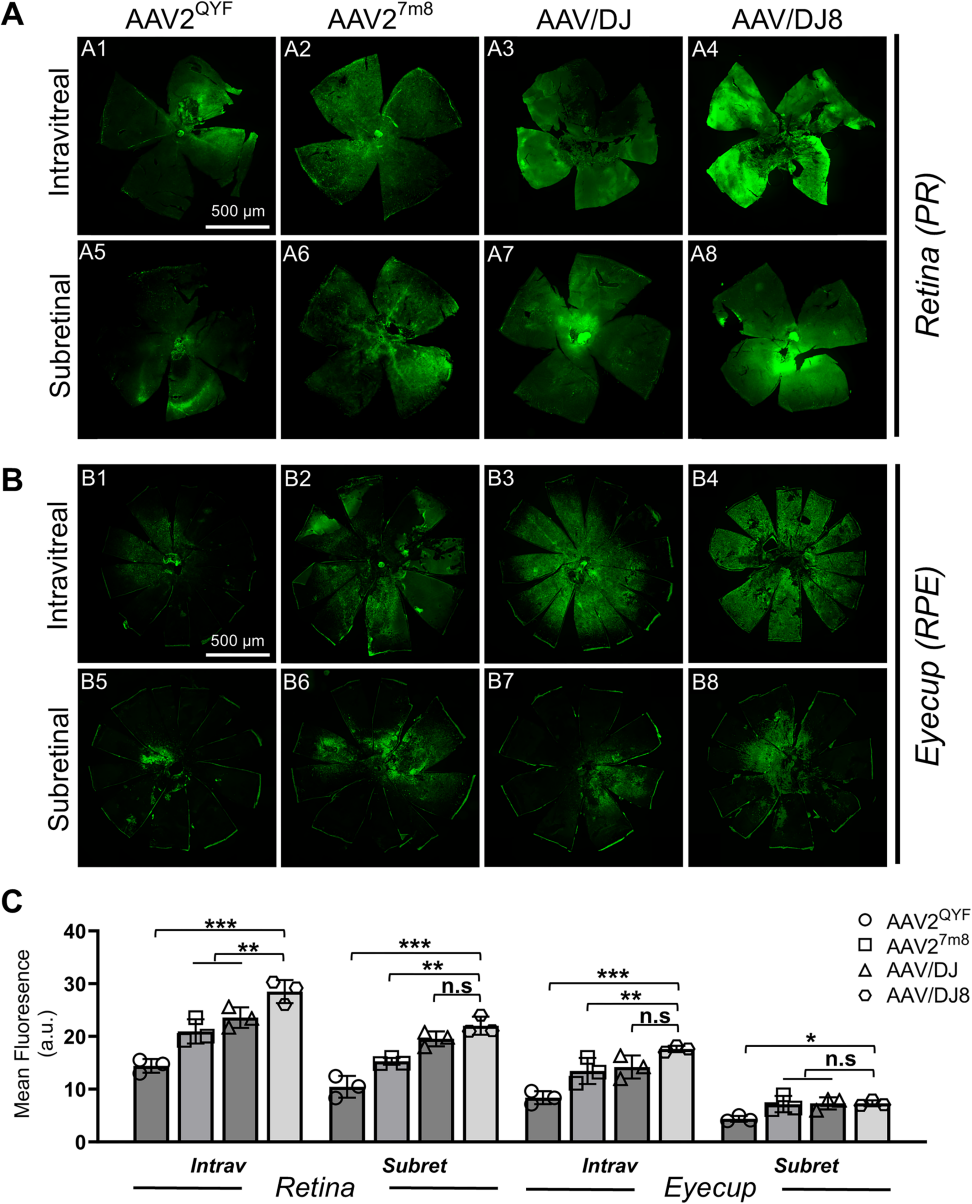
After intravitreal injections, AAV/DJ8 exhibited wide coverage of EGFP expression in the retina. Representative fluorescence images of the distribution of EGFP expression in whole retinas (photoreceptor-side-up) (**A**) and eyecup (RPE-side-up) (**B**) 2 months after intravitreal (A1–A4, B1–B4) and subretinal (A5–A8, B5–B8) injection in the WT mice eye. (**C**) Quantification of EGFP fluorescence intensity in the intravitreal (A1–A4, B1–B4) and subretinal (A5–A8, B5–B8) injected retina (**A**) and eyecup (**B**) 2 months postinjection. Statistical analysis was obtained from four flat mounts per serotype. Results presented as the mean ± SD (one-way ANOVA with Tukey's correction, *P < 0.5, **P < 0.01, ***P < 0.001).

**Figure 4.**
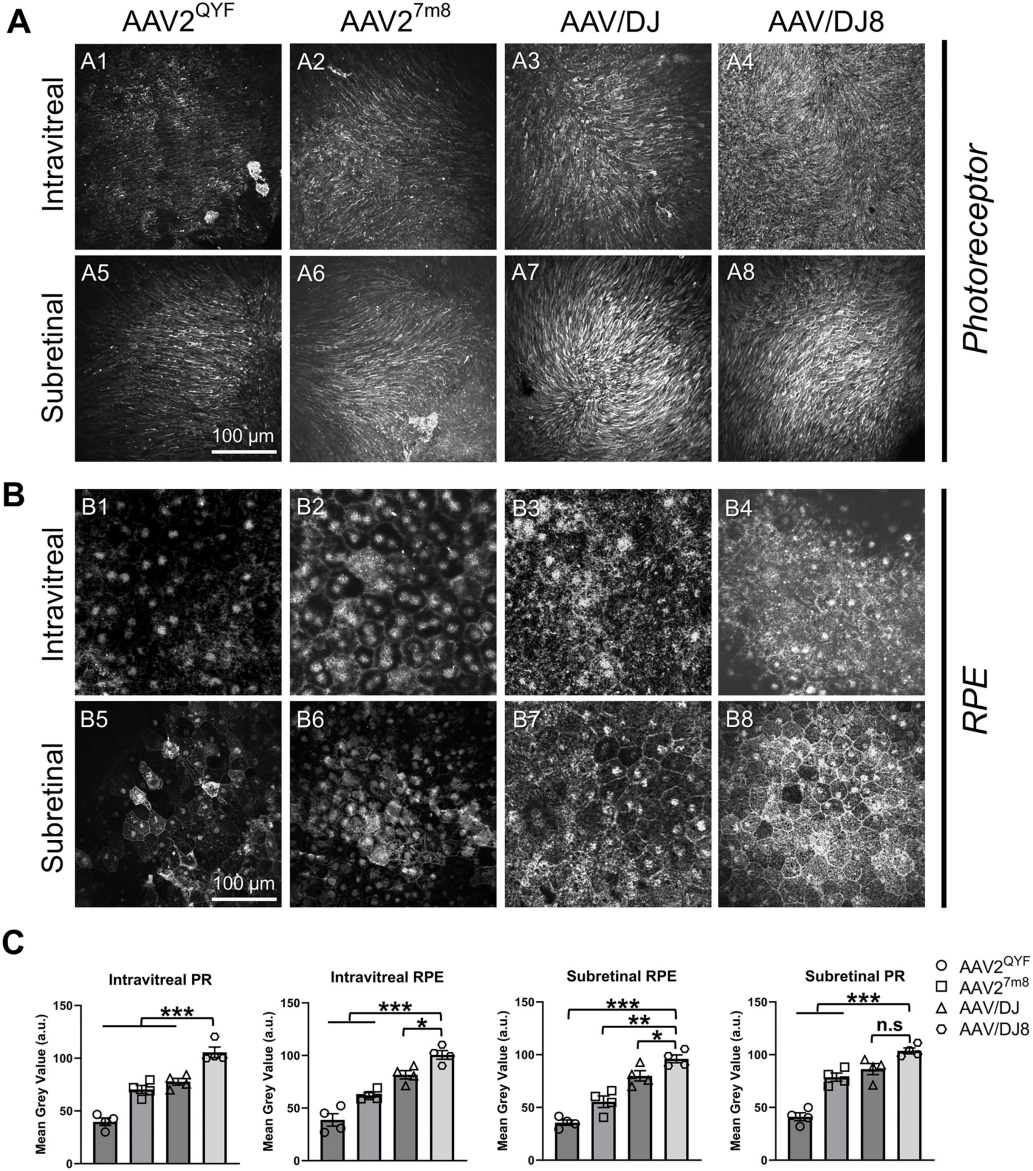
AAV/DJ8 exhibited strong EGFP expression within the mouse PR and RPE cells. Representative high-magnification view of the whole-mounted retina photoreceptor layer (**A**) and RPE layer (**B**) showing strong EGFP expression in PR and RPE cells after intravitreal AAV/DJ8 injection. (**C**) Quantification of mean gray value of EGFP expression in the intravitreal (A1–A4, B1–B4) and subretinal (A5–A8, B5–B8) injected PR (**A**) and RPE (**B**) layer 2 months postinjection. Statistical analysis was obtained from four images of equal area per flat mount and 4 flat mounts/group. Results presented as the mean ± SD (one-way ANOVA with Tukey's correction, *P < 0.5, **P < 0.01, ***P < 0.001).

Two months after administering various viral particles into the eye, we evaluated transgene expression and coverage in the outer retina, specifically the PR and RPE cells. Confocal microscopy revealed that AAV/DJ8 produced the strongest EGFP expression in both the PR and the RPE layers, followed closely by AAV/DJ (Figs. 3A3–A4, 3B3–B4, 3C). In contrast, subretinal injections resulted in more localized EGFP expression around the injection site (Figs. 3A5–A8, 3B5–B8, 3C), which is a recognized limitation of this approach. The AAV2^QYF^ exhibited the lowest EGFP expression in these layers, consistent with fundus imaging and Western blot results (Figs. 2A1, 2A5, 2B1, 2B5, 2E, 2F). At higher magnifications, AAV/DJ8-infected retinas displayed the most intense green fluorescence, while RPE cells from the same retina also showed the highest EGFP signal (Figs. 4A4, 4A8, 4B4, 4B8, 4C). In contrast, AAV2^QYF^ yielded the least expression in both cell types (Figs. 4A1, 4A5, 4B1, 4B5, 4C). Additionally, it is worth noting that the EGFP signal may also originate from intermediate neurons in the retina infected by AAV-viral particles, which we have not accounted for.

### Intravitreally Injected AAV Serotypes Exhibited Serotype-Dependent Tropism for Cells at the Outer Retina

**Figure 5.**
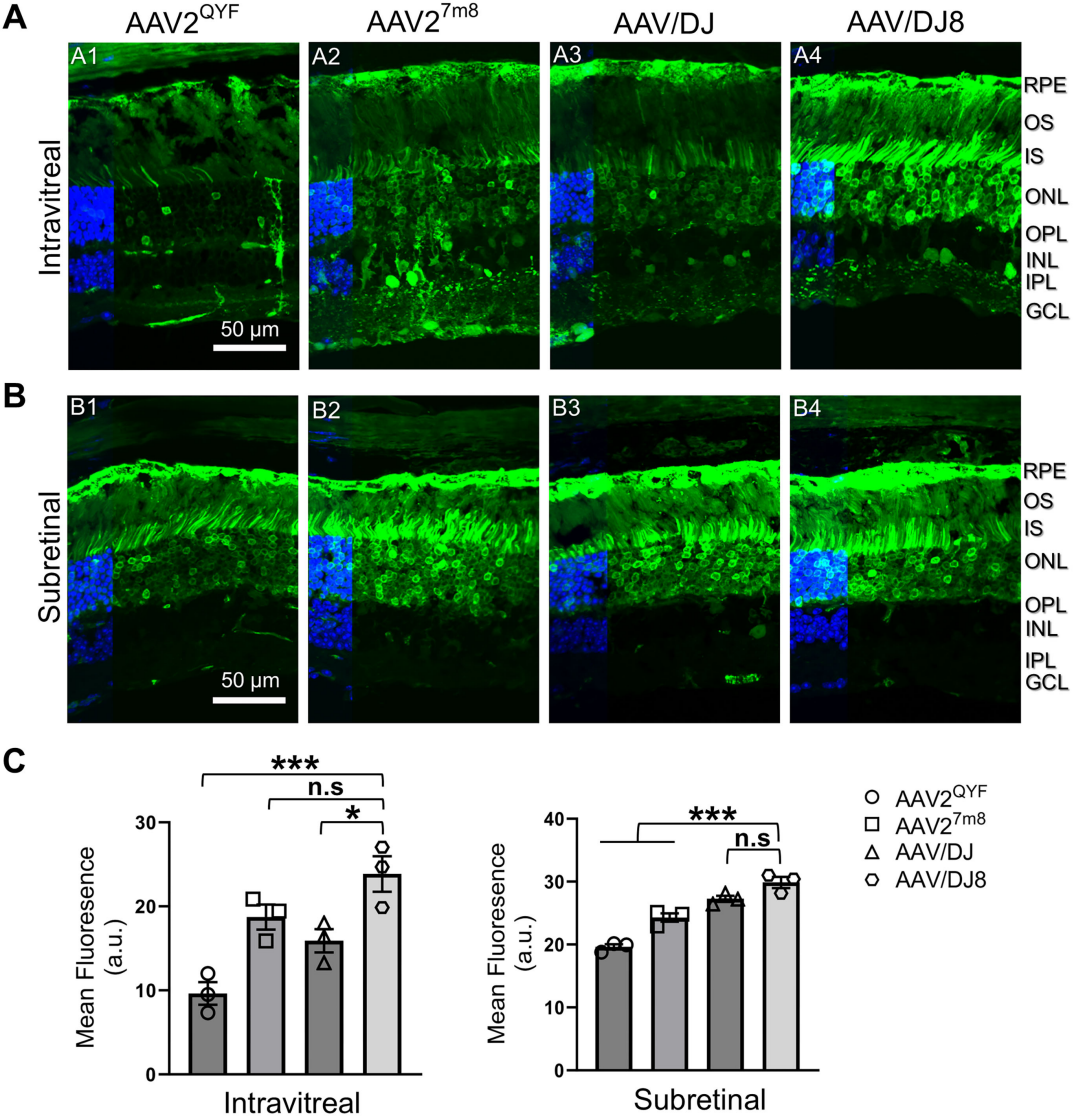
Intravitreally delivered AAV/DJ8 exhibited tropism for PR and RPE cells. Representative EGFP expression counterstained with DAPI (*blue*) images of retina cross sections in intravitreally injected (**A**) and subretinally injected (**B**) mouse eyes of indicated AAV serotypes. *n* = 4 eyes from different mice/group. (**C**) Quantification of EGFP fluorescence intensity in the retina cross-sections of the intravitreally (**A**) and subretinally (**B**) injected mice eyes. Statistical analysis was performed using four images of equal area from mid-peripheral retinal cross sections of different mice. Results presented as the mean ± SD (one-way ANOVA with Tukey's correction, *P < 0.5, **P < 0.01, ***P < 0.001).

Immunofluorescent analysis of EGFP expression on retinal cryosections from the intravitreally injected WT retinas revealed selective tropism among the four rAAV serotypes. Unlike subretinal injections, which showed EGFP expression limited to the PR and the RPE cells (Fig. 5B), intravitreal injections resulted in serotype-dependent EGFP expression (Fig. 5A). AAV/DJ8 showed a strong preference for both PR and the RPE cells (Fig. 5A4), whereas AAV/DJ had a higher affinity for RPE cells than the PRs (Fig. 5A3). EGFP expression in AAV2^QYF^ was limited to some retina cells throughout the retina layer (Fig. 5A2). In contrast, AAV2^7m8^ exhibited transgene expression across the entire retinal cross section (Fig. 5A1), which is consistent with previous reports.^16^^,^^17^

### Functional Changes in the AAV-Injected Eye

**Figure 6.**
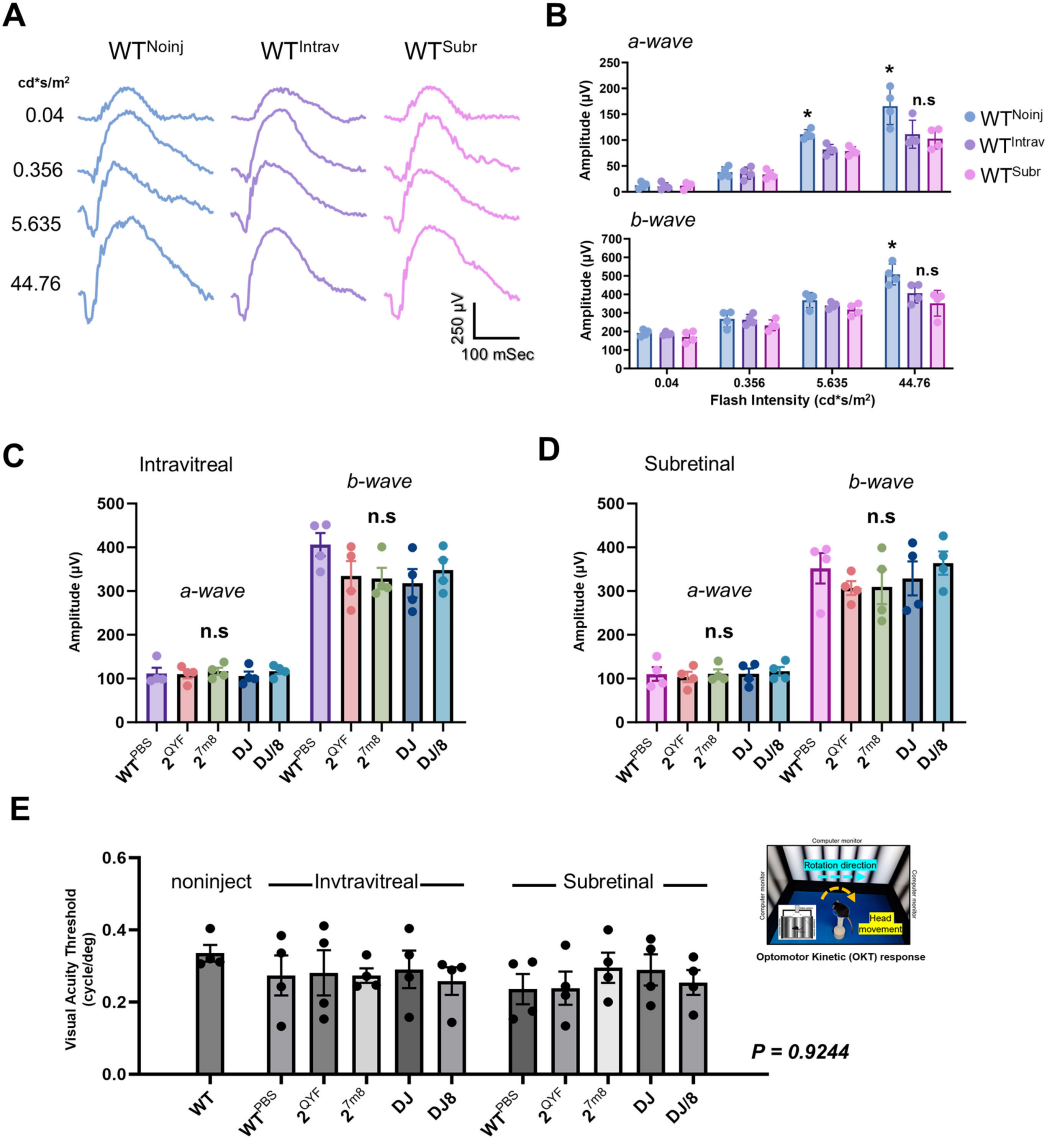
Both intravitreal and subretinal injections caused a decline in ERG amplitudes independent of AAV. Two-month postinjection comparison of visual function via ERG (**A–D**) and OKT (**E**) in mice following intravitreal (**C**) and subretinal (**D**) AAV delivery. Representative ERG waveforms in WT-injected mice eyes (**A**). Scotopic a- and b-wave in intravitreally and subretinally injected WT mice under increasing flash intensities showed reduced ERG amplitudes in injected eyes at higher light intensities (**B**). At 44.76 cd*s/m^2^ flash intensity, no significant difference in ERG amplitudes was detected in AAV-injected eyes, whether via intravitreal (**C**) or subretinal (**D**), compared to the PBS-injected eye. Visual acuity measured in both AAV-injected and noninjected mice eyes shows similar threshold values between the groups (**E**); inset shows a schematic of the experiment. *n* = 4 mice/group. Results presented as the mean ± SD (one-way ANOVA with Tukey's correction, *P < 0.05).

Two months following the initial intraocular injection, ERG was conducted to evaluate retinal function alterations resulting from intraocular delivery of rAAV particles. The findings revealed that PBS-injected eyes exhibited a slight decline in scotopic a- and b-wave ERG amplitudes compared to noninjected control eyes (Fig. 6B), indicating an impact of the injection process on retinal function. Importantly, there was no significant difference in ERG amplitudes between rAAV-injected and PBS-injected eyes, suggesting that AAV-mediated transgene expression did not adversely affect ERG function (Figs. 6C, 6D). This implies that the injection process alone causes a decline in retinal function independent of viral infection, consistent with previous studies.^18^ Visual acuity measured via the optomotor response^29^ showed no significant differences among noninjected, rAAV-injected, and PBS-injected eyes (Fig. 6E).

**Figure 7.**
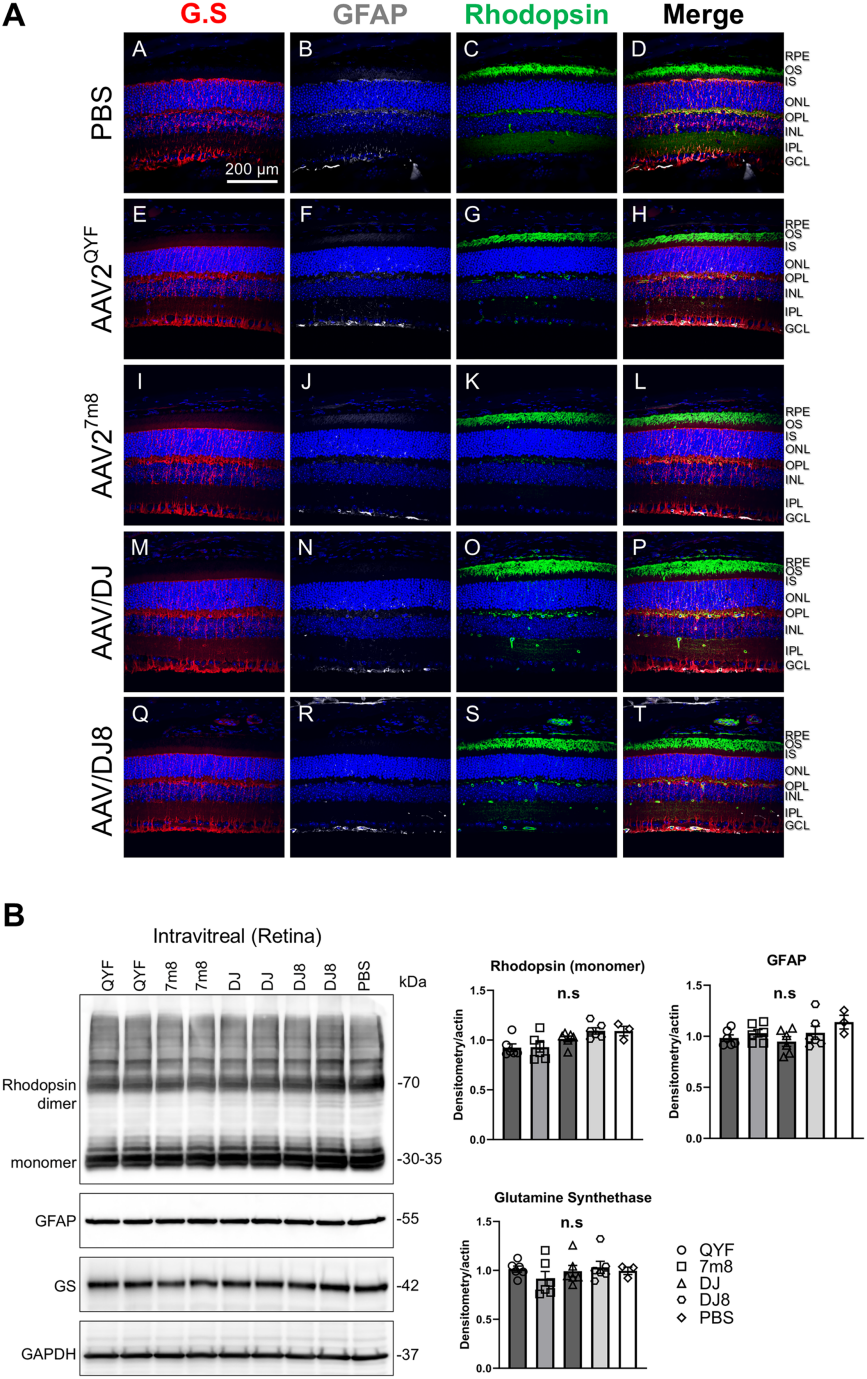
Viral expression did not affect glial reactivity or rhodopsin protein expression in the AAV-injected retinas. Representative histologic images of mid-peripheral retina sections from the mice injected with the respective AAV serotypes and stained with Glutamine Synthetase GS (*red*), Glial Fibrillary Acidic Protein GFAP (*gray*), and Rhodopsin RHO (*green*) 2 months after intravitreal injections (**A**). *n* = 4 eyes/group. Western blot analysis of retinal stress proteins in the 2-month intravitreally injected retinas (**B**). Densitometry quantification reveals no significant difference in protein expression between the AAV- and PBS-injected retinas. Statistical analysis was obtained from three technical repeats. Results presented as the mean ± SD.

The observed decline in ERG may be linked to alterations in rhodopsin (RHO) levels or retina stress responses. Decreases in glutamine synthetase (GS) in Müller glial cells and increases in glial fibrillary acidic protein (GFAP) production are typical markers of retina stress, a hallmark of nearly all retinal diseases.^30^ Immunohistochemical and Western blot analyses were conducted to assess RHO, GS, and GFAP expression in AAV-injected retinal tissues. We observed no significant downregulation of downregulation of GS (Figs. 7A [A, E, I, M, Q], 7B), upregulation of GFAP (Figs. 7A [B, F, J, N, R], 7B), or reduction in PR RHO expression (Figs. 7A [C, G, K, O, S], 7B) in the intravitreally rAAV injected retinas when compared to the control. These results imply that the decline in ERG is likely due to mechanical damage during the injection process rather than alterations in retinal phototransduction or cellular stress.^18^ Indeed, it has been reported that ocular injections can affect ERG responses due to changes in electrolytes, osmolality, and temperature.^31^

## Discussion

This study evaluates the transduction and transgene expression efficiencies of four AAV serotypes (AAV2^QYF^, AAV2^7m8^, AAV/DJ, AAV/DJ8) following intravitreal or subretinal injections. Previous investigations have demonstrated that these serotypes can transduce retinal PR and RPE cells via intravitreal injections. However, variations in factors, such as vector dosage and choice of species for preclinical model evaluations, and transgene cassettes have made direct comparisons challenging.^14^^,^^16^^–^^20^^,^^26^ To address these differences and provide direct comparison of the retinal transduction efficiency of the different AAV serotypes, we standardized all conditions and determined the longitudinal transduction efficiency and EGFP transgene expression in PR and RPE cells for each AAV serotype delivered via intravitreal or subretinal routes. Our findings indicate that intravitreal injections of AAV/DJ8 resulted in superior pan-retinal transduction and EGFP expression in both PR and RPE (Figs. 2D, 2E, 3A4, 3B4), followed by AAV/DJ (Figs. 2D, 2E, 3A3, 3B3) and AAV2^7m8^ (Figs. 2D, 2E, 3A2, 3B2), with AAV2^QYF^ exhibiting the lowest levels of expression (Figs. 2D, 2E, 3A1, 3B1). As expected, subretinal injections resulted in confined EGFP expression primarily surrounding the injection site (Figs. 3A5–A6, 3B5–B6), which is a known limitation of subretinal injections due to the confined lateral movement of the injected material.^32^

All serotypes infected both PR and RPE cells via subretinal delivery; however, AAV2^QYF^ again displayed the lowest transduction efficiency (Fig. 5B), which is consistent with previous reports.^14^^,^^18^^,^^19^ However, intravitreal injections revealed distinct cellular transduction and transgene localization profiles for each serotype, with AAV2^QYF^ showing the least EGFP expression and mostly restricted to the inner retina (Fig. 5A1). As previously reported, AAV^7^^m^^8^ effectively transduced both the inner and outer retina (Fig. 5A2).^16^^,^^17^ AAV/DJ demonstrated a higher tropism for RPE cells compared to PR (Fig. 5A3). Notably, AAV/DJ8 showed high efficiency in transducing PR and RPE cells in the outer retina (Fig. 5A4). The main difference between AAV/DJ and DJ/8 lies in the heparan sulfate proteoglycan binding domain (HBD) in the AAV/DJ capsid. AAV/DJ8 was created by replacing the HBD region with amino acids from a similar site on AAV8, which completely removed the HBD to abolish the heparan-binding properties of AAV/DJ.^26^ We speculate that the removal of HBD in AAV/DJ8 may unintentionally create a novel binding site on the capsid, allowing attachment to an unidentified coreceptor that enhances capsid internalization in PR and RPE cells.

Currently, subretinal delivery is the only effective method for targeting the posterior retina, but intravitreal injections are preferable due to being less invasive and offering broader coverage. The cell tropism, influenced by the AAV vector serotype, is crucial for therapeutic outcomes.^33^ Nevertheless, a significant drawback of intravitreal AAV delivery is the potential for ocular inflammation. Due to the extensive exposure of the vector in the vitreous humor—a compartment that contains immune cells—robust immune responses are observed in patients undergoing this treatment. Therefore, careful considerations must be applied to vector design and dosage control to mitigate the risk of inflammation.

We are aware that other investigators have engineered novel AAV viral serotypes for efficient gene delivery to the human retina that were not tested in this study.^5^^,^^20^ Our study identifies AAV/DJ8 as the most effective serotype for penetrating the outer retina and equally infecting PR and RPE cells via intravitreal delivery. Although AAV/DJ did not outperform AAV/DJ8 in outer retina transduction, it will be worthwhile to examine its efficiencies in nonhuman primate retinas using cell-specific promoters, given AAV/DJ's increased affinity for RPE cells. This increased affinity may be particularly valuable for researchers targeting macular degeneration and vision loss through gene therapy.
